# Supplementary material for: Content-rich biological network constructed by mining PubMed abstracts
Source: BMC Bioinformatics. 2004 Oct 8;5:147. doi: 10.1186/1471-2105-5-147 (PMC528731; doi:10.1186/1471-2105-5-147)
Supplement: Additional File 2 — The original results of the above study (non-essential files are deleted to keep the file size under the limit set by BMC bioinformatics). [file 1471-2105-5-147-S2.bz2 › chilibotAdditionalFile2/dip30/399ID9662041E9953/html/EGFR_BTC.html]

 


 **EGFR** and **BTC** 
  
Found 65 abstracts in PubMed, retrieved 30.  
 

 What does Google say? 
 PDF only 
| .edu only 

---

**Interactive relationship** (e.g. stimulation, inhibition, etc)

**Stimulatory relationship**- Betacellulin  [ **BTC** ]  also acts as a ligand for ErbB  [ **EGFR** ]  4 to stimulate its kinase activity in both homo and hetero dimers.  Ref: 10882304 J Mammary Gland Biol Neoplasia, 1997
- The results are consistent with a model in which GLP 1 increases PI 3 kinase activity and enhances beta cell proliferation via transactivation of the  **EGFR**  that would require the proteolytic processing of membrane anchored  **BTC**  or other EGF like ligands.  Ref: 12502502 Diabetes, 2003
- Recombinant rat  **BTC**  stimulated the proliferation of mouse Balb c 3T3 fibroblasts and competed for binding to the ErbB1  [ **EGFR** ]  receptor in a dose dependent manner analogous to that of  **BTC**  purified from natural sources.  Ref: 11564606 J Mol Endocrinol, 2001
**Neutral relationship**- Heparin binding epidermal growth factor HB EGF and betacellulin  [ **BTC** ]   **BTC**  are activating ligands for EGF receptor  **EGFR**  ErbB1  [ **EGFR** ]  and ErbB4.  Ref: 12773386 EMBO J< MedlineTA>EMBO J, 2001
- **BTC** , acting through EGF R erbB 1  [ **EGFR** ] , is important for the differentiation of beta cells.  Ref: 12399441 Endocrinology, 2002
- Betacellulin  [ **BTC** ]  and heregulin NDF alpha strikingly colocalized with  **EGFr**  and erbB3 receptors in the granular layer and in a declining gradient from the granular zone to the basal layer, respectively.  Ref: 12768307 Arch Dermatol Res< MedlineTA>Arch Dermatol Res, 2002
- **BTC**  binds and activates ErbB 1  [ **EGFR** ]  and ErbB  [ **EGFR** ]  4 homodimers and is further characterised by its unique ability to activate all possible heterodimeric ErbB  [ **EGFR** ]  receptors.  Ref: 10940639 Int J Biochem Cell Biol, 2000
- Surprisingly, overexpression of the ErbB  [ **EGFR** ]  receptors even when combined with treatment with their ligands EGF, transforming growth factor alpha, betacellulin  [ **BTC** ] , neuregulins failed to protect the MCF 7 cells from cell death induced by either tumor necrosis factor TNF or serum starvation.  Ref: 10797281 Int J Cancer, 2000
- Among four EGF receptor  **EGFR**  family members, the  **EGFR**  was solely activated by EGF, heparin binding EGF HB EGF, transforming growth factor alpha TGF alpha, epiregulin ER, and betacellulin  [ **BTC** ]   **BTC** , resulting in induction of phenotypic modulation of SMCs.  Ref: 11181057 Biochem Biophys Res Commun, 2001
- Betacellulin  [ **BTC** ]   **BTC**  purified from mouse beta cell tumor betaTC 3 is a new member of the epidermal growth factor EGF family which can bind receptor tyrosine kinase, EGF receptor erbB1  [ **EGFR** ]  and erbB4.  Ref: 10724350 Endocr J, 1999
- Betacellulin  [ **BTC** ]   **BTC**  belongs to the epidermal growth factor EGF family of peptide ligands that are characterized by a six cysteine consensus motif EGF motif that forms three intra molecular disulfide bonds, crucial for binding the ErbB  [ **EGFR** ]  receptor family.  Ref: 12031500 Biochim Biophys Acta, 2002
- AG1478 decreased the expression of genes for amphiregulin, HB EGF and betacellulin  [ **BTC** ] , showing that  **EGFR**  activation induces up regulation of the  **EGFR**  ligands.  Ref: 11133810 Carcinogenesis, 2000

**Non-interactive relationship** (e.g. studied together, co-existance, homology, etc.)

- While much is known about the ErbB  [ **EGFR** ]  receptor binding characteristics of  **BTC**  and its effect on a variety of cultured cells under different conditions, the challenge that lies ahead is to determine the role of  **BTC**  in vivo.  Ref: 10940639 Int J Biochem Cell Biol, 2000
- This hypothesis was supported by the finding that the growth inhibitory effect of marimastat was directly related to its ability to prevent the release of major c erbB  [ **EGFR** ]  ligands including transforming growth factor alpha, betacellulin  [ **BTC** ]  and heregulin beta1 from HNSCC.  Ref: 12124801 Int J Cancer, 2002
- Using semi quantitative reverse transcription PCR, we showed that all three c erbB  [ **EGFR** ]  ligands up regulated VEGF A mRNA all isoforms and VEGF C  **BTC**  max at 1 10 nM.  Ref: 11235991 Clin Exp Metastasis, 2000
- Betacellulin  [ **BTC** ]   **BTC**  belongs to the epidermal growth factor EGF family of peptide ligands that are characterised by a six cysteine consensus motif that forms three intra molecular disulfide bonds crucial for binding the ErbB  [ **EGFR** ]  receptor family.  Ref: 10940639 Int J Biochem Cell Biol, 2000
- Genomic structure and promoter characterization of the gene encoding the ErbB  [ **EGFR** ]  ligand betacellulin  [ **BTC** ] .  Ref: 12031500 Biochim Biophys Acta, 2002
- Pretreatment with effective concentrations of ErbB1  [ **EGFR** ]  inhibitor did not suppress  **BTC**  induced kinase phosphorylation.  Ref: 12475887 FASEB J, 2003
- We demonstrate that mRNAs for the 6 major c erbB  [ **EGFR** ]  ligands, namely, epidermal growth factor EGF, transforming growth factor alpha TGF alpha, betacellulin  [ **BTC** ]   **BTC** , heparin binding epidermal growth factor like growth factor HB EGF, amphiregulin AR and heregulin HRG, are expressed in a large panel of HNSCC cell lines.  Ref: 11072245 Int J Cancer, 2000
- The data herein demonstrate that heregulin or betacellulin  [ **BTC** ] , but not EGF, promotes the rapid translocation of ErbB  [ **EGFR** ]  2, the heterodimerization partner for ErbB  [ **EGFR** ]  4, to the same detergent insoluble fraction in T47 D mammary carcinoma cells.  Ref: 11439355 Oncogene, 2001
- Little is known about the regulative role of the four other widely expressed epidermal growth factor receptor  [ **EGFR** ]  ligands, heparin binding EGF, amphiregulin, betacellulin  [ **BTC** ]  and cripto in the gastric mucosa.  Ref: 11218236 Scand J Gastroenterol, 2001
- Moreover, immunohistochemical staining revealed that that  **BTC** , ErbB1  [ **EGFR** ] , and ErbB4 are upregulated in the plaques of human atherosclerotic coronary arteries.  Ref: 12869389 Circ ResCirc Res, 2001
- **BTC**  and EP also present the distinctive feature that they activate all possible heterodimeric ErbB  [ **EGFR** ]  receptors.  Ref: 12111392 J Mol Model , 2002
- Both the c Src inhibitor PP1 and the  **EGFR**  specific inhibitor AG1478 blocked GLP 1 induced 3 H thymidine incorporation in INS 832 13 cells as well as in isolated rat islets, while only AG1478 inhibited the proliferative action of betacellulin  [ **BTC** ]   **BTC** , an  **EGFR**  agonist.  Ref: 12502502 Diabetes, 2003
- ICR62 also reversed the effects of all three erbB  [ **EGFR** ]  ligands TGF alpha,  **BTC**  and HRG beta1 on VEGF A, VEGF C and VEGF D expression.  Ref: 11235991 Clin Exp Metastasis, 2000
- A soluble erbB  [ **EGFR** ]  4 binding fusion protein totally inhibited the effects of NRG 4 but not of  **BTC** .  Ref: 12399441 Endocrinology, 2002
- **BTC**  induced phosphorylation of all four EGF receptors present on HTASMCs ErbB1  [ **EGFR** ] , ErbB2, ErbB3, and ErbB4.  Ref: 12869389 Circ ResCirc Res, 2002
- We then explored the regulatory roles of three major ligands with different selectivity of binding to c erbB  [ **EGFR** ]  receptors namely transforming growth factor alpha TGF alpha, betacellulin  [ **BTC** ]   **BTC**  and heregulin beta1 HRG beta1 on VEGF A, B, C and D expression in selected HNSCC lines.  Ref: 11235991 Clin Exp Metastasis, 2000
- Reverse transcription polymerase chain reaction was performed on each sample with primers for rat epidermal growth factor, epidermal growth factor receptor  [ **EGFR** ]  ErbB  [ **EGFR** ] , heparin binding epidermal like growth factor, hepatocyte growth factor, hepatocyte growth factor receptor, keratinocyte growth factor, betacellulin  [ **BTC** ] , amphiregulin, and neuregulin alpha.  Ref: 12148846 Laryngoscope, 2002
- **EGFR**  mRNA was detected in all tumours, transforming growth factor alpha TGFalpha mRNA levels were similar to those in normal bladder urothelia or were decreased and mRNA levels for amphiregulin, heparin binding epidermal growth factor like factor HB EGF and betacellulin  [ **BTC** ]  were significantly higher than those in normal urothelia.  Ref: 11133810 Carcinogenesis, 2000
